# Identifying novel circadian rhythm biomarkers for diagnosis and prognosis of melanoma by an integrated bioinformatics and machine learning approach

**DOI:** 10.18632/aging.205961

**Published:** 2024-06-20

**Authors:** Yi Xu, Churuo Zeng, Jie Bin, Hua Tang, Wei Li

**Affiliations:** 1 Department of Plastic Surgery, Second People’s Hospital of Hunan Province, Changsha, Hunan, China

**Keywords:** melanoma, circadian rhythm, biomarker, prognostic model, bioinformatics, machine learning

## Abstract

Melanoma is a highly malignant skin tumor with poor prognosis. Circadian rhythm is closely related to melanoma pathogenesis. This study aimed to identify key circadian rhythm genes (CRGs) in melanoma and explore their potential as diagnostic and prognostic biomarkers. Microarray data of melanoma tissues and normal skins were obtained. Differentially expressed genes were identified and weighted gene co-expression network analysis (WGCNA) was performed to screen hub genes associated with melanoma. By overlapping hub genes with known CRGs, 125 melanoma-related CRGs were identified. Functional enrichment analysis revealed these CRGs were mainly involved in circadian rhythm and other cancer-related pathways. Three machine learning algorithms including LASSO regression, support vector machine-recursive feature elimination (SVM-RFE), and random forest were utilized to select key CRGs. Six CRGs (ABCC2, CA14, EGR3, FBXW7, LDHB, and PSEN2) were identified as key CRGs for melanoma diagnosis and prognosis. Diagnostic values of key CRGs were evaluated by ROC analysis in training and validation sets. Prognostic values of key CRGs were assessed by survival analysis and a multivariate Cox regression prognostic model was constructed. The prognostic model could effectively stratify melanoma patients into high- and low-risk groups with significantly different survival. A nomogram integrating clinical variables and risk score was built to predict 3-, 5- and 10-year overall survival of melanoma patients. In summary, six CRGs were identified as key genes associated with melanoma pathogenesis and may serve as promising diagnostic and prognostic biomarkers. The prognostic model and nomogram could facilitate personalized prognosis evaluation of melanoma patients.

## INTRODUCTION

Melanoma is a highly invasive skin tumor, known for its rapid growth and strong metastatic characteristics. Melanoma mainly occurs in young and middle-aged people. In recent years, its incidence has unfortunately been steadily rising, and its rate of increase is faster than any other solid tumor. According to the World Health Organization, about 287,000 people worldwide are diagnosed with melanoma every year, and nearly 60,000 die from it [1, 2]. Melanoma has become a serious public health problem, imposing great economic burden on society. Due to the lack of effective early diagnosis and screening methods, many patients are diagnosed and treated at a late stage, resulting in poor prognosis. Statistically, the 5-year survival rate for metastatic melanoma patients is only 4.6% [3]. In terms of treatment, early-stage melanoma is primarily managed by surgical resection, however, it is prone to recurrence; while late-stage melanoma is mainly treated with chemotherapy. In recent years, some progress has been made in immunotherapy using immune checkpoint inhibitors and cytotoxic T lymphocytes, though the overall therapeutic outcome remains suboptimal [4].

The pathogenesis of melanoma is complex, involving multiple genetic and environmental factors. Strong and intermittent sun exposure, ultraviolet radiation, the number of nevi in the host, genetic susceptibility, etc. are risk factors for melanoma [5]. Abnormal expression of many tumor suppressor genes or oncogenes is an essential cause of melanoma. For instance, the tumor suppressor gene BRAF is closely associated with the onset, progression, and prognosis of melanoma [5]. Therefore, exploring the molecular characteristics of melanoma and identifying new diagnostic and prognostic biomarkers are crucial for understanding the pathological mechanisms of melanoma, developing new effective diagnostic and treatment methods, and improving the clinical outcomes of patients.

Circadian rhythm is an endogenous, approximately 24-hour periodic biological clock in organisms that regulates many physiological and behavioral activities [6]. In recent years, research has found that circadian rhythm is closely related to the pathogenesis, development, prognosis and therapeutic response of melanoma. Circadian rhythm disturbance is a risk factor for melanoma [7–10]. During the pathogenesis of melanoma, abnormal expression or genetic variation of circadian rhythm genes (CRGs) occurs, and these abnormalities are important causes leading to melanoma pathogenesis and poor prognosis [10]. Although some CRGs have been found to be closely related to melanoma pathogenesis and prognosis, researchers’ attention on the circadian rhythm mechanism of melanoma is still insufficient. Therefore, comprehensive mining of the relationship between melanoma and circadian rhythm genes is of great significance.

In recent years, bioinformatics and machine learning technologies have been widely applied to the analysis of gene and protein expression profiles, which helps rapidly and accurately screen disease biomarkers, construct disease prognostic models and explore pathological mechanisms. Therefore, this study utilized bioinformatics and machine learning technologies to mine CRGs and molecular mechanisms closely related to melanoma, so as to provide a reference for the diagnosis, prognostic evaluation and potential therapeutic target discovery of melanoma.

## MATERIALS AND METHODS

### Data download and preprocessing

Firstly, 4 melanoma-related transcriptomic datasets (GSE15605, GSE114445, GSE46517 and GSE65904 datasets) were downloaded from GEO database (https://www.ncbi.nlm.nih.gov/geo/), and 1 melanoma-related gene expression profile data was obtained from TCGA database (https://tcga-data.nci.nih.gov/tcga/). A total of 1471 circadian rhythm related genes (CRGs) were obtained from MSigDB database (https://www.gsea-msigdb.org/gsea/msigdb/) and Genecards database (https://www.genecards.org/) (Supplementary Table 1). Then, preprocessing was performed on each expression profile data. The R software package “limma” was used for background correction, median normalization and gene symbol conversion of each dataset.

GSE15605 and GSE114445 datasets were based on GPL570 platform (Affymetrix Human Genome U133 Plus 2.0 Array). GSE15605 dataset contained 16 normal skin samples and 46 primary melanoma samples, and GSE114445 dataset contained 6 normal skin samples and 15 primary melanoma samples. GSE46517 dataset was based on GPL96 platform (Affymetrix Human Genome U133A Array), containing 8 normal skin samples and 31 primary melanoma samples. GSE65904 data was based on GPL10558 platform (Illumina HumanHT-12 V4.0 expression beadchip), containing tumor tissue samples from 214 melanoma patients. TCGA-melanoma dataset included tumor tissue samples from 458 melanoma patients.

In this study, GSE15605 and GSE114445 datasets were combined as a new expression profile as the training set, which was used for the selection of key CRGs in melanoma. Separate GSE15605, GSE114445 and GSE46517 datasets were used as validation sets to validate the expression of key CRGs and evaluate the diagnostic ability of key CRGs. The TCGA-melanoma cohort was used as the training set for prognostic evaluation and prognostic model construction of key CRGs. GSE65904 dataset was used as the validation set for verification of the prognostic model.

### Screening of melanoma-related circadian rhythm genes (CRGs)

### Dataset merging

Firstly, GSE15605 and GSE114445 datasets were merged as the training set for screening melanoma-related key CRGs. To eliminate batch effects between different datasets, batch correction was performed on the merged dataset using the “sva” package in R software. Then the box plots, density plots of gene expression and Uniform Manifold Approximation and Projection (UMAP) analysis were performed to validate the effect of batch correction.

### Differential expression analysis

Differential expression analysis was performed using R software package “limma”. Differential expression genes (DEGs) between melanoma and normal skin samples in the training set were screened with the threshold of “|logFC|>1.2, *P*<0.05”.

### Weighted gene co-expression network analysis (WGCNA)

The R package “WGCNA” was used to perform weighted gene co-expression network analysis (WGCNA) on the expression matrix of the above DEGs. Specifically, we first constructed an adjacency matrix to describe the association strength between nodes. Then, the optimal soft thresholding power β was chosen to transform the adjacency matrix into topological overlap matrix (TOM), making the constructed network follow scale-free topology and be closer to real biological networks. Next, module partitioning analysis was performed using hierarchical clustering and dynamic tree cut algorithm to determine gene co-expression modules. The module eigengenes (MEs) of each gene module were then calculated and the connectivity between different modules was analyzed. The correlation between each module and clinical trait (melanoma and normal skin) was then calculated. Finally, gene significance (GS) and module membership (MM) were calculated, and hub genes closely related to melanoma pathogenesis were screened in the modules with GS>0.6, MM>0.6 and *P*<0.05.

### Screening of melanoma-related CRGs

The hub genes obtained from the WGCNA network were compared with the CRGs downloaded from the MSigDB database to identify the common genes, which were the melanoma-related CRGs that affect the occurrence and development of melanoma.

### Functional enrichment analysis and protein-protein interaction (PPI) analysis

To explore the potential biological functions and signaling pathways of circadian rhythm genes (CRGs) in melanoma pathogenesis, we performed functional enrichment analysis on melanoma-related CRGs using DAVID database. Specifically, Gene Ontology (GO) functional annotation and Kyoto Encyclopedia of Genes and Genomes (KEGG) functional enrichment analysis were performed on the above melanoma-related CRGs. For GO functional annotation, we selected “Gene Ontology” as the analysis type in the DAVID database and chose “Biological Process”, “Molecular Function”, and “Cellular Component” as the annotation types. We selected the species as human and submitted the list of CRGs. For KEGG functional enrichment analysis, we used the DAVID database and selected “KEGG Pathway” as the analysis type. Finally, we visualized the analysis results.

To further evaluate the role of circadian rhythm mechanisms in melanoma pathogenesis, we performed Gene Set Enrichment Analysis (GSEA), a gene set-based enrichment analysis method that identifies significant differences in biological functions and signaling pathways between two biological conditions. Specifically, we obtained Gene Ontology (GO) biological processes gene sets, including ENTRAINMENT OF CIRCADIAN CLOCK, CIRCADIAN REGULATION OF GENE EXPRESSION, CIRCADIAN RHYTHM, and REGULATION OF CIRCADIAN RHYTHM, as well as the KEGG gene set CIRCADIAN RHYTHM, from the MSigDB database (https://www.gsea-msigdb.org/gsea/msigdb) as reference gene sets. We then used GSEA software (version 4.0.1) to determine the expression differences of these circadian rhythm-related biological functions and signaling pathways between melanoma and normal skin tissues.

In addition, the protein-protein interaction (PPI) network between these melanoma-related CRGs was further constructed. First, these melanoma-related CRGs were uploaded to STRING database (https://string-db.org/) for protein-protein interaction (PPI) analysis. Then Cytoscape software (version 3.7.2) was used to construct the PPI network and analyze the topological parameters of nodes in the network.

### Identification of key CRGs associated with melanoma

We utilized 3 machine learning algorithms including support vector machine-recursive feature elimination (SVM-RFE) analysis, random forest analysis and LASSO regression analysis to screen key genes from the above melanoma-related CRGs.

SVM-RFE is an embedded feature selection approach that ranks features based on their importance [11]. It repetitively trains SVM classifier models and removes the feature with smallest ranking criterion each time until all features are ranked. Here, we utilized SVM-RFE algorithm in R package “e1071” to rank the melanoma-related CRGs by their importance. The CRGs were recursively eliminated until the optimal SVM model with high classification accuracy was obtained. The remaining CRGs were identified as key features.

Random forest is an ensemble supervised learning technique constructed by multiple decision trees [12]. It can estimate the importance of features by permutation and assess prediction error using out-of-bag samples. Here, we used the random forest algorithm in R package “randomForest” to select and rank CRGs by their importance. The top 20 ranked CRGs by importance were selected as key genes under random forest analysis.

LASSO regression is a regularization technique that performs feature selection and regularization to enhance prediction accuracy and interpretability. It penalizes the absolute size of regression coefficients, effectively reducing model complexity and avoiding overfitting [13]. Here, we constructed a LASSO regression model based on melanoma tissue and normal skin tissue samples using R package “rms”. By tuning the regularization parameter λ, LASSO regression selects important features and shrinks the coefficients of unimportant features to zero. In this study, we identified the melanoma-related key CRGs by selecting the features with non-zero coefficients in the LASSO model when λ was set as 0.04.

The commonly selected feature genes by the 3 machine learning methods were identified as key CRGs associated with melanoma.

### Expression and ROC analysis of key CRGs

Based on the training set (merged GSE15605 and GSE114445 datasets) and validation sets (GSE15605, GSE114445 and GSE46517 datasets), Wilcoxon rank-sum test was performed to investigate the expression of key CRGs in melanoma tissues and normal skin tissues, respectively.

Then, to further validate the accuracy of key CRGs selection and evaluate the diagnostic value of key CRGs as melanoma biomarkers, receiver operating characteristic (ROC) analysis was performed on the training and validation sets using the “pROC” package in R software. The diagnostic power was reflected by the area under ROC curve (AUC) value. AUC>0.7 indicated that key CRGs had good ability to distinguish melanoma from normal skin tissues.

### Prognostic analysis of key CRGs

Firstly, we performed Kaplan-Meier survival curve analysis for each key CRG using the ‘survival’ package in R software based on the TCGA database which integrated transcriptomic data and clinical information of melanoma patients, in order to assess their impacts on the prognosis of melanoma patients. Then, multivariate Cox regression analysis with stepwise variable selection was utilized to construct a prognostic model with key CRGs significantly associated with prognosis of melanoma patients, and determine the relative coefficient of each feature gene. At each step, the most significant CRG was added into the model. CRGs that became non-significant were removed from the final prognostic model. For each patient, the CRGs risk score was calculated as follows:


$$\text{Risk}\;\text{Score}=\sum_{i=0}^{n}Coefficient_{\left(mRNAi\right)}\times {\text{Expression}}_{\left(mRNAi\right)}$$

According to the median value of risk scores, melanoma patients in the training set (TCGA cohort) and validation set (GSE65904 dataset) were classified into high- and low-risk groups. Kaplan-Meier survival analysis was performed to evaluate the survival difference between high- and low-risk groups. ROC analysis at 3, 5 and 10 years was performed using the R package “survivalROC” to evaluate the sensitivity and specificity of the prognostic model.

Then, to further predict the prognosis of melanoma patients, we constructed a nomogram. The nomogram is a statistical prediction model that generates a visualization tool for estimating individualized probabilities of a clinical outcome, such as patient survival, based on a combination of important variables. Here, we analyzed the relationship between different clinical pathological parameters including stage, Tumor (T), Node (N), Metastasis (M) classification and risk score using Spearman’s rank correlation test. Benjamini-Hochberg procedure was applied for multiple testing correction. Subsequently, integrating clinical features (age, sex, stage, T/N/M classifications) and risk score, univariate and multivariate Cox regression analyses were performed. Using the rms package in R software, all independent prognostic parameters were utilized to construct a nomogram to predict 3-, 5- and 10-year overall survival of melanoma patients. The predictive ability of the nomogram was validated by calibration analysis and ROC analysis.

## RESULTS

### Screening of melanoma-related CRGs

### Dataset merging

**Figure 1:**
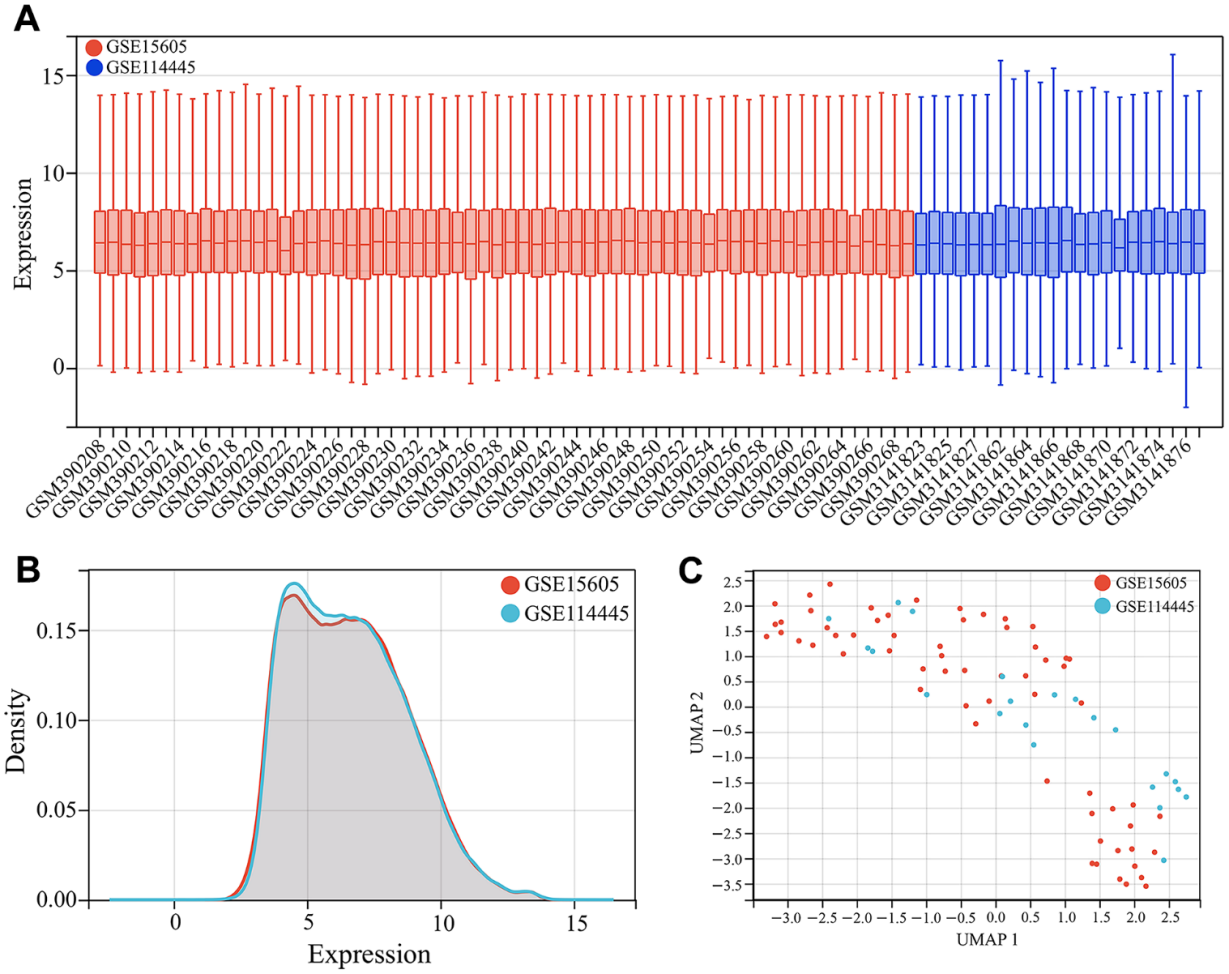
**Dataset merging.** (**A**) Gene expression boxplots of the merged dataset after batch correction. (**B**) Gene expression density plots of the merged dataset after batch correction. (**C**) Uniform Manifold Approximation and Projection (UMAP) analysis of the merged dataset after batch correction.

**Figure 2:**
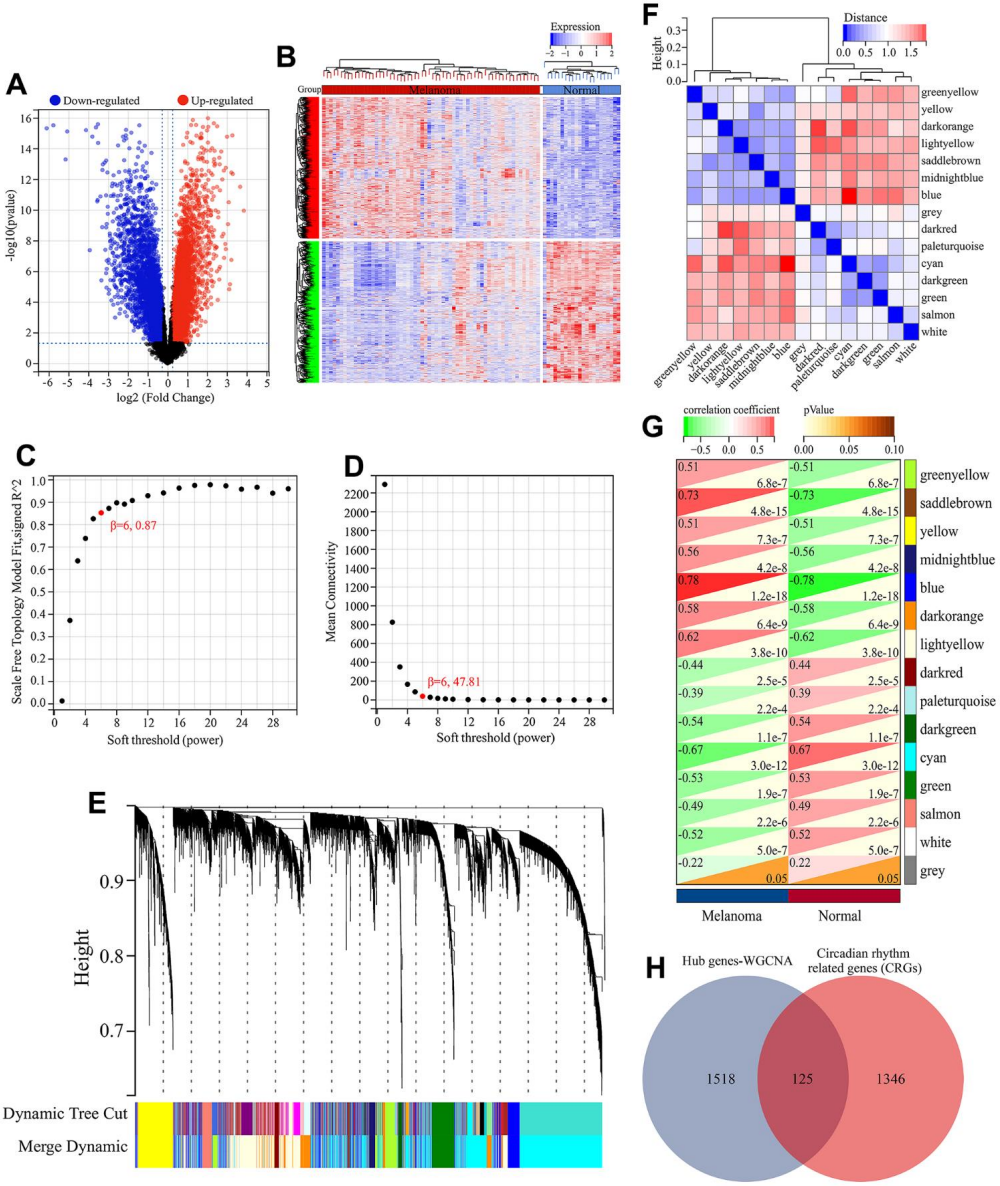
**Screening of melanoma-related CRGs.** (**A**) Volcano plot of differential expression analysis. (**B**) Heatmap of differential expressed genes. (**C**) Relationship between scale-free topology and soft thresholding power. (**D**) Average connectivity’s dependence on soft threshold power levels. (**E**) Hierarchical clustering dendrogram displaying distinct co-expression modules as individual colors. The bicolored rows beneath the tree indicate the initial and combined modules. (**F**) Cluster dendrogram of module eigengenes (MEs) and adjacency heatmap of MEs. (**G**) Correlation heatmap illustrating the association between module eigengenes (MEs) and clinical characteristics. (**H**) Screening of melanoma-related CRGs. The overlapping genes between hub genes in WGCNA and CRGs obtained from MSigDB database were defined as melanoma-related CRGs.

We first merged GSE15605 and GSE114445 datasets as the training set for screening melanoma-related key CRGs. After batch correction, we plotted the gene expression boxplots and density plots, and performed Uniform Manifold Approximation and Projection (UMAP) analysis to validate its effects. The gene expression boxplots and density plots showed that the data distributions across different datasets became consistent after removing batch effects (Figures 1A, 2B). The UMAP analysis results showed that each sample in GSE15605 and GSE114445 datasets was randomly and discretely distributed (Figure 1C). The results indicated that batch correction could effectively eliminate batch effects between different datasets, making the merged dataset more consistent and reliable.

### Screening of melanoma-related CRGs

Through differential expression analysis, 8725 DEGs were identified between melanoma tissues and normal skin tissues (Figure 2A, 2B). These DEGs were further analyzed by WGCNA to screen genes associated with melanoma. In WGCNA, the optimal soft thresholding power β was chosen as 6 (Figure 2C, 2D). Then modules were cut using hierarchical clustering and DynamicTreeCut algorithm. With the maximum module distance set as 0.25, highly similar modules were merged, and eventually 15 independent gene modules were generated, namely greenyellow, saddlebrown, yellow, midnightblue, blue, darkorange, lightyellow, darkred, paleturquoise, darkgreen, cyan, green, salmon, white and grey (Figure 2E). We then analyzed the independence of these gene co-expression modules. As shown in Figure 2F, the distances between modules were all greater than 0.25, indicating these gene modules had good independence. Next, we calculated the correlation between all modules and clinical trait (melanoma and normal skin). The results showed that except grey module, all other modules were significantly correlated with melanoma (Figure 2G). Therefore, these melanoma-related modules were selected for further screening genes closely related to melanoma pathogenesis. With the threshold of GS>0.6, MM>0.6 and *P*<0.05, 1643 hub genes were identified from the co-expression network of these gene modules (Supplementary Table 2). These hub genes were considered to be closely related to melanoma pathogenesis.

Finally, by comparing CRGs and these hub genes, 125 common genes were identified and considered as CRGs that affect melanoma pathogenesis and development (Figure 2H).

### Functional enrichment analysis and protein-protein interaction (PPI) analysis

**Figure 3:**
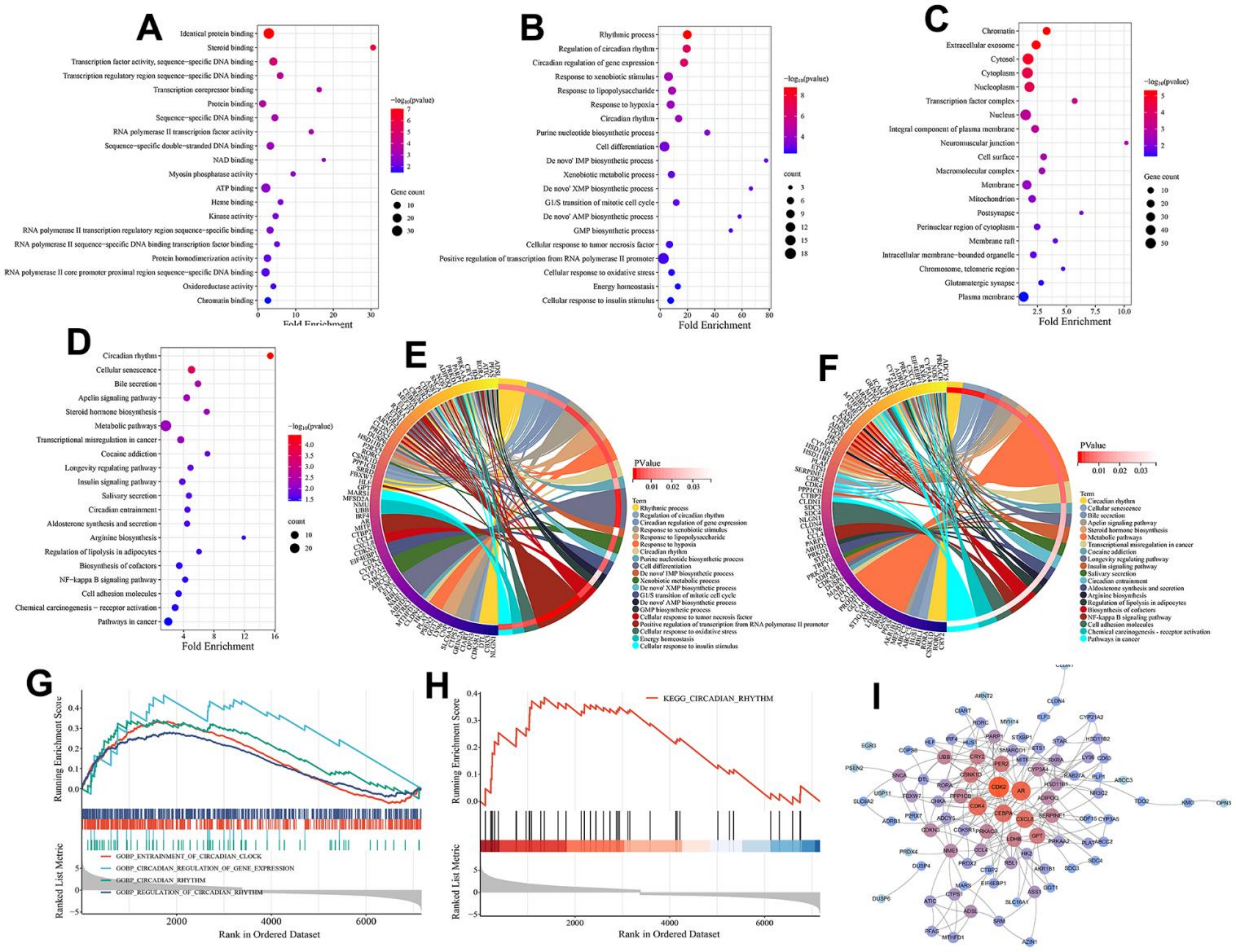
**Functional enrichment analysis and PPI analysis.** (**A**) Molecular function (MF) analysis (top 20 terms ranked by statistical significance). (**B**) Biological processes (BP) analysis (top 20 terms ranked by statistical significance). (**C**) Cellular component (CC) analysis (top 20 terms ranked by statistical significance). (**D**) KEGG pathway enrichment analysis (top 20 terms ranked by statistical significance). (**E**) The interaction of BPs and their associated CRGs. (**F**) The interaction of KEGG signaling pathways and their associated CRGs. (**G**) Gene set enrichment analysis (GSEA) on BPs associated with circadian rhythm. (**H**) Gene set enrichment analysis (GSEA) on a KEGG pathway associated with circadian rhythm. (**I**) The PPI network of 125 melanoma-related CRGs. Nodes are colored by degree, with redder colors indicating higher degree centrality within the network.

Here, the biological functions and signaling pathways related to circadian rhythm mechanism in melanoma were identified. GO functional enrichment analysis of the 125 melanoma-associated circadian rhythm genes (CRGs) revealed their involvement in molecular functions (MFs) including identical protein binding, steroid binding, transcription factor activity, sequence-specific DNA binding, and transcription corepressor binding (Figure 3A). These CRGs were enriched in biological processes (BPs) such as rhythmic process, regulation of circadian rhythm, circadian regulation of gene expression, and response to xenobiotic stimulus (Figure 3B, 3E), and were associated with various cellular components (CCs) including chromatin, extracellular exosome, cytosol, cytoplasm, and nucleoplasm (Figure 3C). KEGG analysis showed that besides directly regulating the circadian rhythm pathway, these melanoma-related CRGs may also indirectly participate in the circadian mechanisms of melanoma by modulating other signaling pathways such as cellular senescence, bile secretion, apelin signaling pathway, insulin signaling pathway, and NF-kappa B signaling pathway (Figure 3D, 3F).

**Figure 4:**
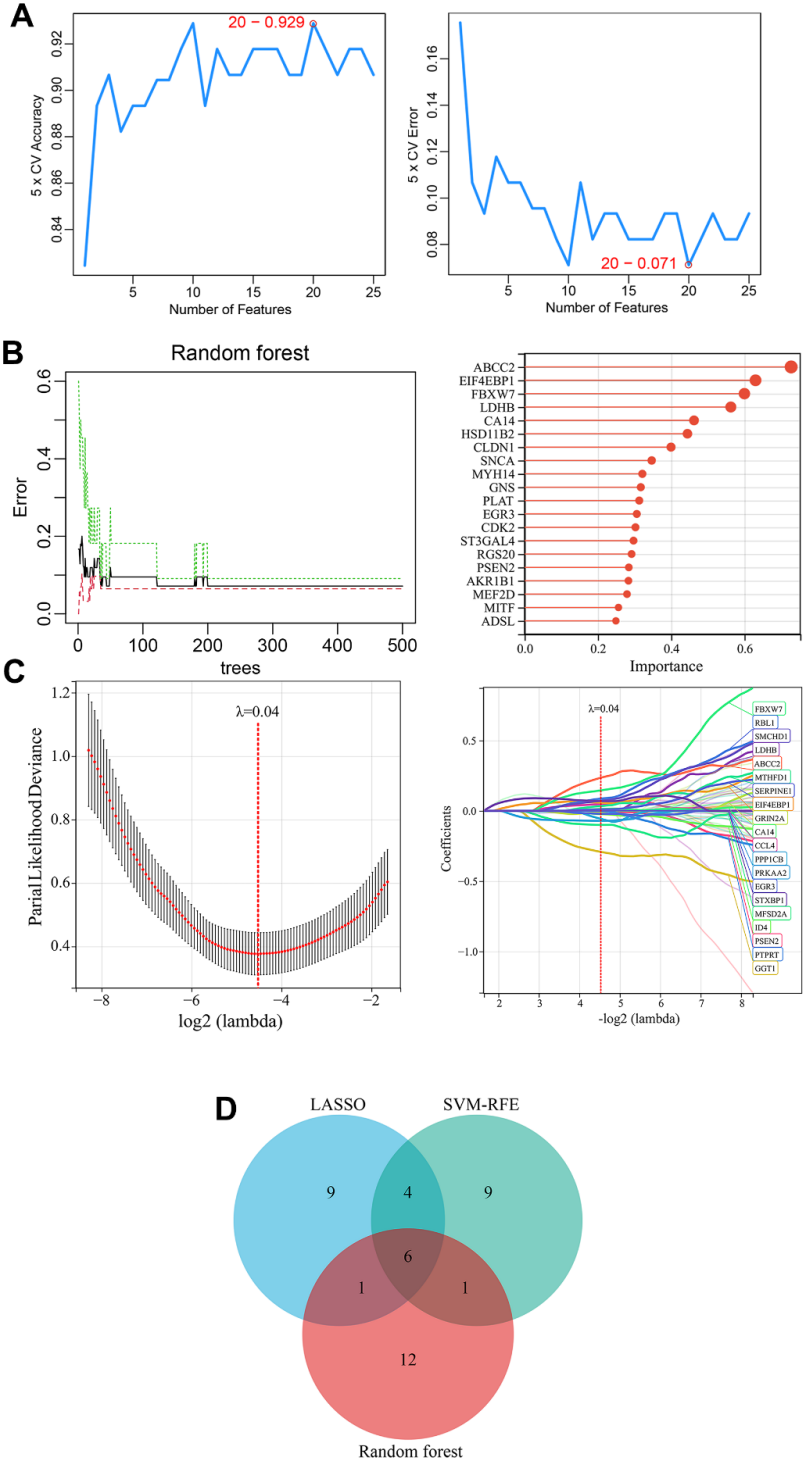
**Identification of melanoma-related key CRGs by machine learning approaches.** (**A**) Feature CRG selection using SVM-RFE algorithm. (**B**) Importance ranking of CRGs using a random forest algorithm. The top 20 CRGs ranked by importance were selected as feature genes. (**C**) Selection of melanoma-associated feature genes using LASSO regression model. (**D**) Identification of melanoma-related key CRGs. The overlapping feature genes (CRGs) from the three machine learning approaches were defined as melanoma-related key CRGs.

GESA analysis further highlighted the significant roles of circadian-associated biological processes and pathways in melanoma. As shown in Figure 4G, 4H, four circadian-related BPs (GOBP ENTRAINMENT OF CIRCADIAN CLOCK, GOBP REGULATION OF CIRCADIAN RHYTHM, GOBP CIRCADIAN RHYTHM, GOBP CIRCADIAN REGULATION OF GENE EXPRESSION) and one KEGG pathway (KEGG CIRCADIAN RHYTHM) were significantly upregulated in the tumor tissues. In addition, we evaluated the interactions between these melanoma-related CRGs by establishing a PPI network. The results showed these melanoma-related CRGs were highly interconnected. Genes including CDK2, AR, CDK4, CEBPA, and CXCL8 occupied central positions in the network control, implying their potential regulatory interactions with numerous genes in the PPI network (Figure 3I).

### Identification of key CRGs associated with melanoma

Three machine learning algorithms including LASSO regression, SVM-RFE, and random forest were utilized to select key CRGs from the 125 melanoma-related CRGs. Firstly, SVM-RFE analysis identified 20 feature CRGs whose classification model achieved 92.9% accuracy in evaluating melanoma samples (Figure 4A). Then, random forest algorithm was used to select and rank CRGs by importance. The top 20 CRGs by importance ranking were selected as key genes under random forest analysis (Figure 4B). Further, a LASSO regression model was constructed based on melanoma tissue and normal skin tissue samples. When λ was set as 0.04, 20 feature CRGs were selected and the LASSO model constructed using these CRGs could accurately distinguish melanoma from normal skin (Figure 4C). Finally, by comparing the feature genes identified by the three machine learning approaches, six common key CRGs were identified as melanoma-related key CRGs, including ABCC2, CA14, EGR3, FBXW7, LDHB, and PSEN2 (Figure 4D).

### Expression and ROC analysis of key CRGs

**Figure 5:**
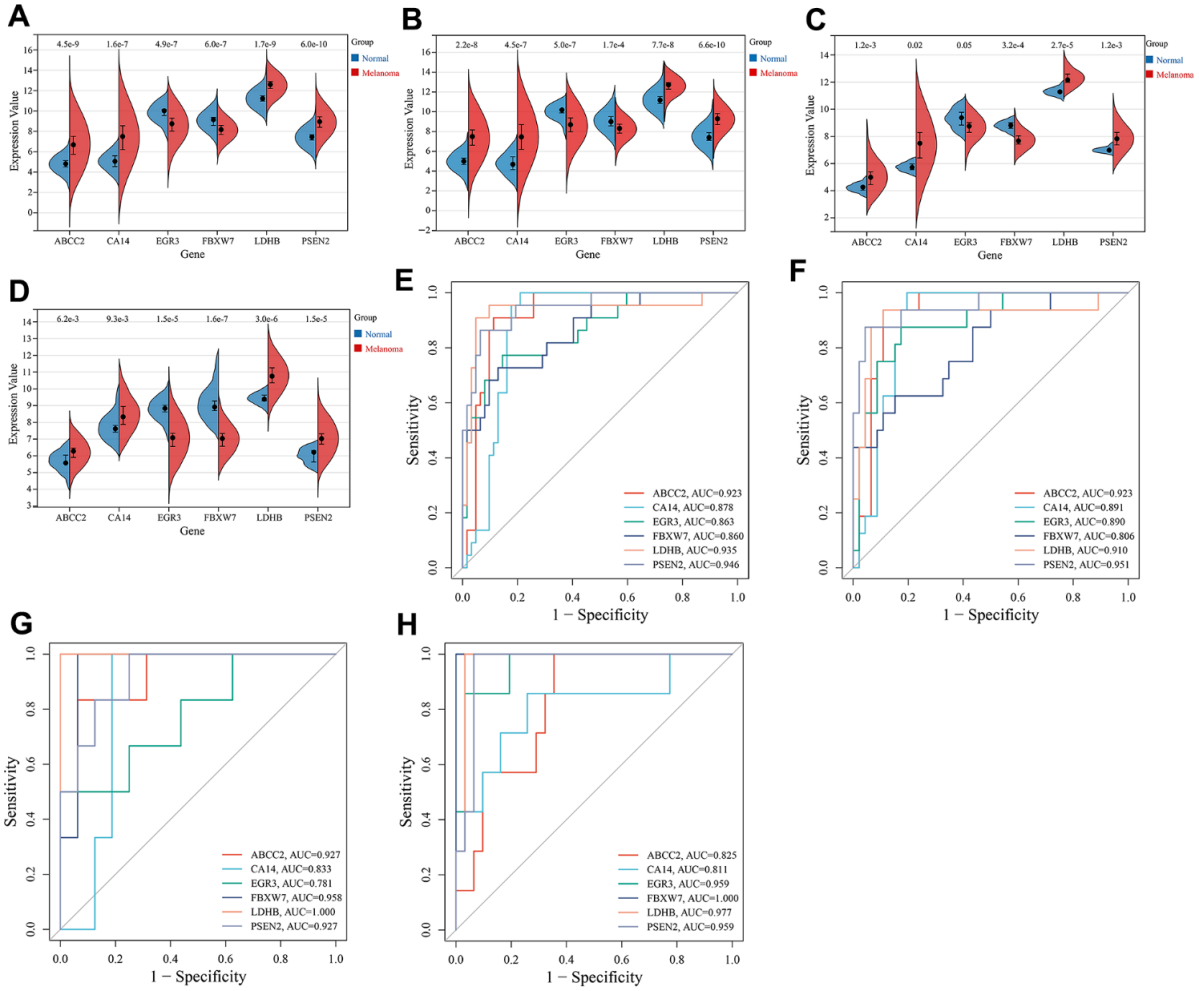
**Expression and ROC analysis of key CRGs.** (**A**) Expression comparison of key CRGs between melanoma and normal tissues in the training set (combined GSE15605 and GSE114445 datasets). (**B**) Expression comparison of key CRGs between melanoma and normal tissues in GSE15605 dataset. (**C**) Expression comparison of key CRGs between melanoma and normal tissues in GSE114445 dataset. (**D**) Expression comparison of key CRGs between melanoma and normal tissues in GSE46517 dataset. (**E**) ROC curves of key CRGs in the training set. (**F**) ROC curves of key CRGs in GSE15605 dataset. (**G**) ROC curves of key CRGs in GSE114445 dataset. (**H**) ROC curves of key CRGs in GSE46517 dataset.

The expression of key CRGs was examined in the training set (combined GSE15605 and GSE114445 datasets) and validation sets (GSE15605, GSE114445 and GSE46517 datasets) to investigate their expression in melanoma versus normal skin tissues, as well as evaluate their diagnostic value as melanoma biomarkers. As shown in Figure 5A–5D, compared to normal skin tissues, 4 key CRGs (ABCC2, CA14, LDHB, PSEN2) were significantly upregulated while 2 key CRGs (EGR3, FBXW7) were significantly downregulated in melanoma tissues. ROC analysis demonstrated that in each dataset, all key CRGs had an AUC>0.7, indicating these key CRGs have good ability to distinguish melanoma from normal skin tissues (Figure 5E–5H).

### Prognostic analysis of key CRGs

**Figure 6:**
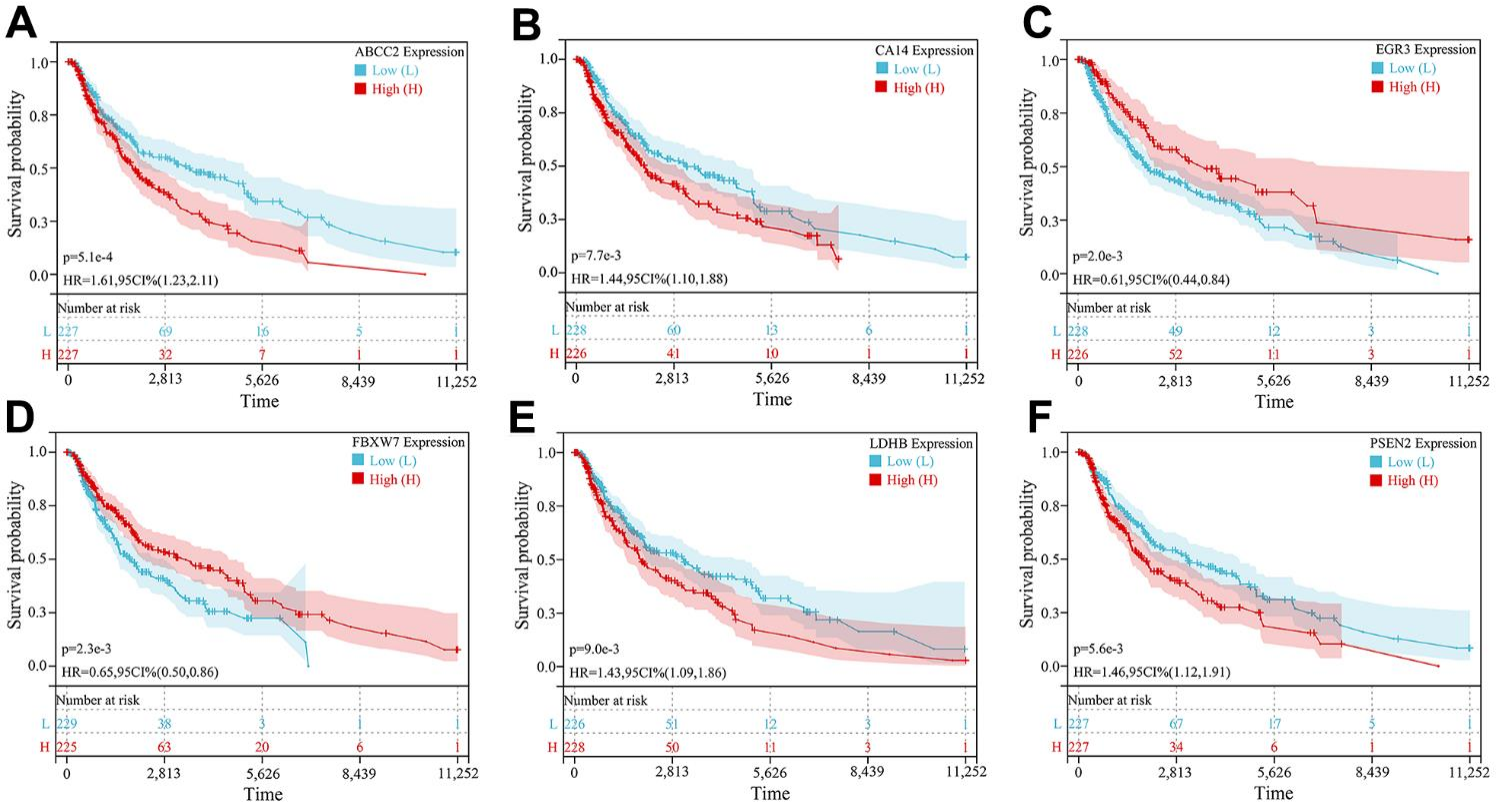
**Kaplan-Meier survival curve analysis of 6 key CRGs.** (**A**–**F**) Kaplan-Meier survival curves of (**A**) ABCC2, (**B**) CA14, (**C**) EGR3, (**D**) FBXW7, (**E**) LDHB, and (**F**) PSEN2, respectively.

Further prognostic analysis of key CRGs was performed based on the TCGA dataset. Kaplan-Meier survival curve analysis showed all key CRGs were closely associated with prognosis in melanoma patients. Specifically, high expression of 4 key CRGs (ABCC2, CA14, LDHB, PSEN2) was significantly correlated with poor prognosis in melanoma patients (HR>1, *P*<0.05), while low expression of 2 key CRGs (EGR3, FBXW7) was significantly associated with poor prognosis (HR<1, *P*<0.05) (Figure 6A–6F).

**Figure 7:**
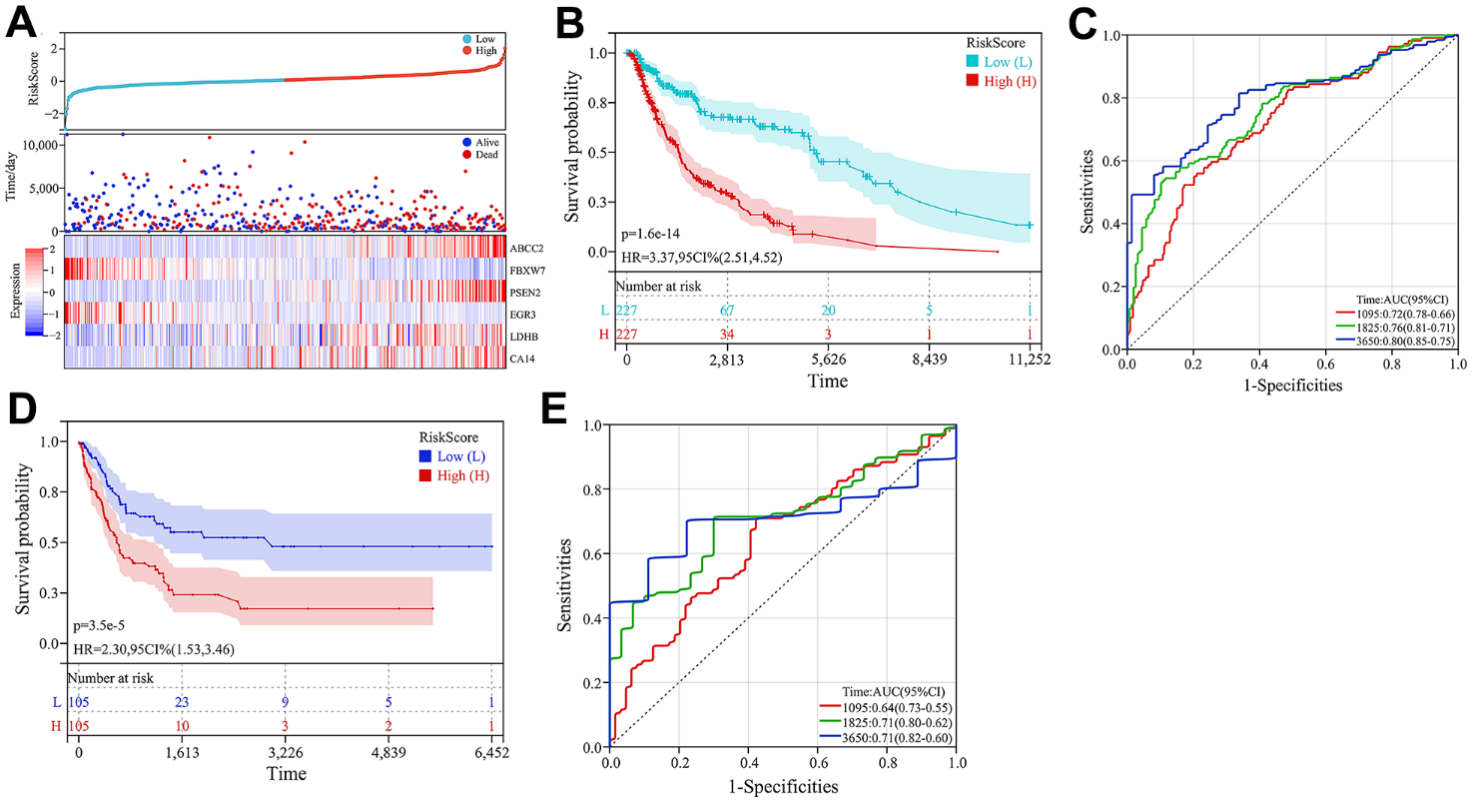
**Construction and validation of the prognostic model.** (**A**) Heatmap of key CRG expression and related risk score ranking and survival status distribution of patients. (**B**) Kaplan-Meier curves comparing high- and low-risk groups in the TCGA-melanoma cohort. (**C**) ROC curves of the prognostic model for predicting 3-, 5- and 10-year overall survival in TCGA-melanoma cohort. (**D**) Kaplan-Meier survival curve analysis in GSE65904 dataset. (**E**) ROC curves of the prognostic model for predicting 3-, 5- and 10-year overall survival in GSE65904 dataset.

Subsequently, multivariate Cox regression analysis was conducted on the 6 prognosis-related key CRGs in the TCGA-melanoma cohort to construct a prognostic risk model based on their weighted regression coefficients. The risk score for each patient was calculated as follows: Risk Score = (0.0320×ABCC2) + (0.0005×CA14) + (-0.0146×EGR3) + (-0.1551×FBXW7) + (0.0010×LDHB) + (0.0073×PSEN2). According to the median value of risk scores, the TCGA cohort was classified into high-risk and low-risk groups. As shown in Figure 7A, the expression levels of the 6 key CRGs were displayed along with the risk score distribution and survival status of all patients. Kaplan-Meier analysis revealed that patients in the high-risk group had significantly poorer prognosis compared to the low-risk group (Figure 7B). The ROC results showed that this prognostic model achieved AUC of 0.72, 0.76 and 0.80 for predicting 3-, 5- and 10-year overall survival of patients, respectively (Figure 7C). Similarly, risk scores were calculated for patients in the validation cohort (GSE65904), and no significant difference in prognosis was found between the high- and low-risk groups (Figure 7D). The risk score model achieved AUC of 0.64, 0.71 and 0.71 for predicting 3-, 5- and 10-year overall survival (Figure 7E). These results indicated this prognostic model had good predictive performance.

**Figure 8:**
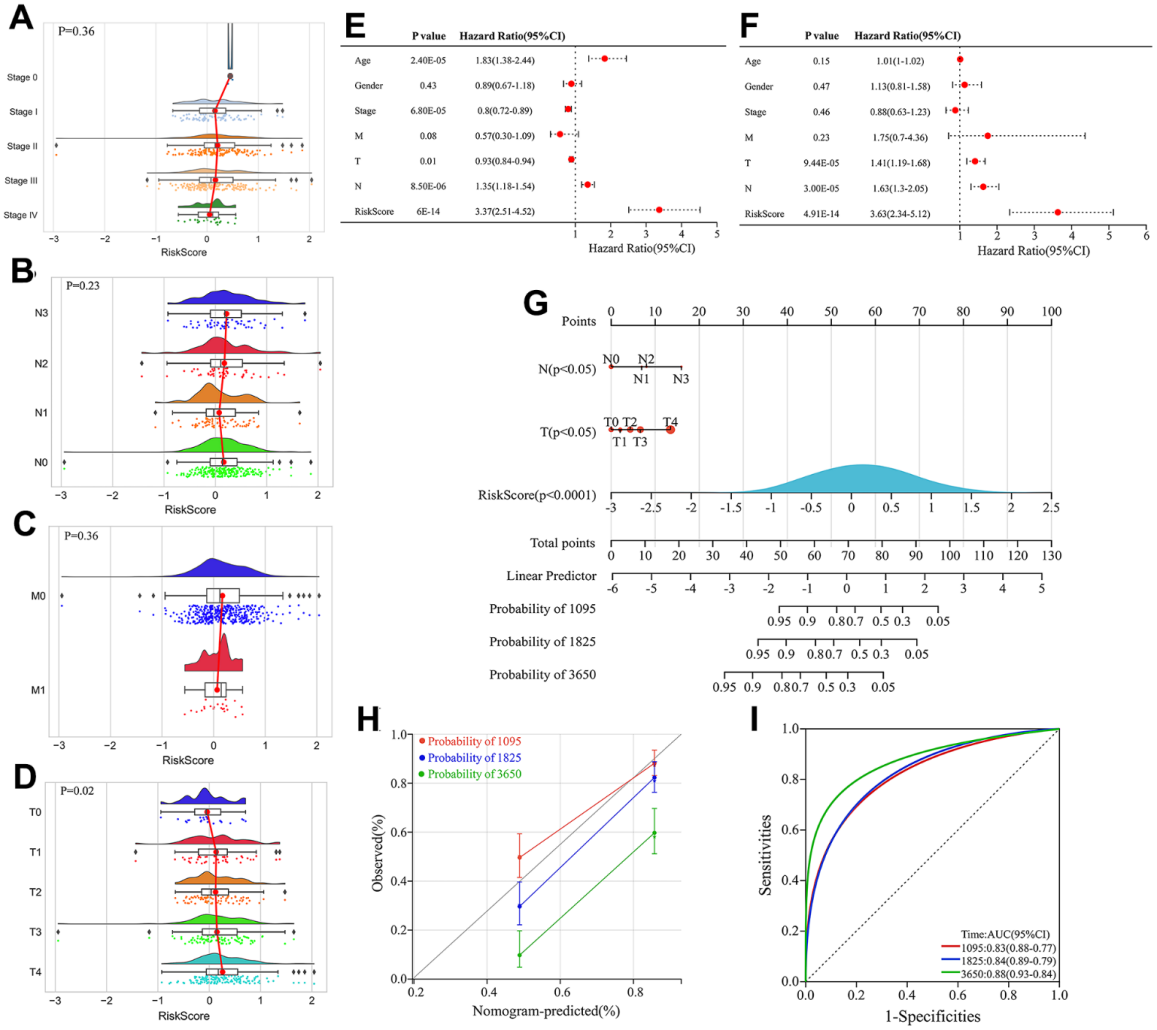
**Construction and evaluation of the nomogram.** (**A**–**D**) Correlation between stage, Node (N), Metastasis (M) classification, Tumor (T) and risk score. (**E**) Univariate Cox regression analyses of clinical features and risk score. (**F**) Multivariate Cox regression analyses of clinical features and risk score. (**G**) Construction of the nomogram by integrating T classification, N classification and risk score to predicting 3-, 5- and 10-year overall survival probabilities in melanoma patients. (**H**) Calibration curve of the nomogram depicting the agreement between nomogram-predicted and observed 3-, 5-, and 10-year overall survival. (**I**) ROC curves evaluating the sensitivity and specificity of the nomogram for predicting 3-, 5-, and 10-year overall survival of melanoma patients.

We next investigated the correlation between the risk score and clinicopathological features of patients in the TCGA cohort, including age, gender, stage, T classification, N classification and M classification. The risk score exhibited significant positive correlations with advanced T classification (*P*=0.02) (Figure 8A–8D).

Univariate and multivariate Cox regression analyses integrating clinical variables (age, sex, stage, T, N and M classification) and risk score identified T classification, N classification and risk score as independent prognostic factors (Figure 8E, 8F). Therefore, a nomogram was constructed by integrating T classification, N classification and risk score to predict patients’ survival probability (Figure 8G). The calibration curve verified high prediction accuracy of the nomogram, which achieved AUC of 0.83, 0.84 and 0.88 for predicting 3-, 5- and 10-year overall survival, respectively (Figure 8H). Compared with risk score alone, the nomogram had higher AUC values, indicating that the risk score had better predictive potential when combined with clinical factors.

## DISCUSSION

The circadian system comprises a central clock in the suprachiasmatic nucleus of the brain and peripheral clocks in tissues throughout the body. The suprachiasmatic nucleus functions as the master regulator, synchronizing circadian rhythms in peripheral clocks. Light is the major cue that entrains the suprachiasmatic nucleus to the light-dark cycle via retinal signals. At a molecular level, circadian rhythms are controlled by transcriptional-translational feedback loops involving circadian rhythms genes (CRGs, also called clock genes) such as Period, Cryptochrome, Bmal1 and Clock [6]. These CRGs regulate their own expression levels in cycles of approximately 24 hours. They also control the expression of other genes involved in many biological processes, such as cell cycle transition, DNA damage response and cell death process [14]. Epidemiological and experimental evidence indicates that circadian disruption leads to various diseases including mental illness, metabolic syndrome, obesity and cancer, directly linked to abnormal expression of clock genes [15, 16]. As the primary barrier to the external environment, the skin is directly influenced by cues like light and plays an important role in regulating circadian rhythms. Studies have demonstrated sustained expression of circadian clock genes in the skin [10]. As a common skin malignancy, melanoma is closely associated with circadian mechanisms [7–9]. Circadian disruption impairs immune responses and DNA repair in melanocytes, increasing melanoma risk [7]. In melanoma, disturbed circadian rhythms promote abnormal proliferation and metastasis, facilitating melanoma development [8]. In addition, circadian rhythm disorder can also induce remodeling of melanoma-related immune microenvironment to facilitate tumor growth [9]. Currently, abnormal circadian gene expression and mutations are thought to underlie the relationship between circadian mechanisms and melanoma [17]. For example, Lengyel et al. found circadian genes per1, per2, clock, and cry1 significantly downregulated in melanoma tissue compared to normal skin [18]. The clock gene RORA is also significantly downregulated in melanoma and its low expression correlates with poor prognosis [19]. Compared to healthy individuals, melanoma patients show significant RORA gene mutations. Benna C et al. [17] identified two specific RORA single nucleotide polymorphisms (SNP) may influence melanoma susceptibility. Another study found two RORA SNPs (RORA rs782917 G > A, RORA rs17204952 C > T) associated with poor melanoma prognosis [20]. As a key circadian regulator in melanoma, downregulation of BMAL1 facilitates melanoma growth and metastasis, and compromises patient survival [21, 22]. These findings indicate circadian clock genes have excellent potential as diagnostic and prognostic biomarkers in melanoma and may serve as therapeutic targets.

In our study, we systematically investigated circadian rhythm gene (CRG) expression, functions, and impacts on melanoma development and prognosis. We identified 125 CRGs associated with melanoma. These genes were mainly enriched in circadian rhythm related pathways and biological processes, and involved in other signaling pathways including Cellular senescence, NF-kappa B signaling pathway, etc. As discussed above, cellular senescence, NF-kappa B signaling pathway and other signaling pathways are also important mechanisms in melanoma. Like the CRGs discussed above, signaling pathways such as Cellular senescence and NF-kappa B signaling pathway are important mechanisms in melanoma. For example, studies have shown that the continuous accumulation of senescent melanocytes, fibroblasts, keratinocytes and immune cells in skin can promote melanoma pathogenesis and affect treatment, which is related to immune system aging [23]. Senescence inhibition therapy reduced tumor volumes and extended survival in a mouse melanoma model [23]. NF-kappa B signaling pathway plays an important role in melanoma, promotes melanoma cell proliferation, survival, invasion, and microenvironment formation [24]. Notably, circadian rhythm disruption can activate cellular senescence [25] and NF-kappa B signaling pathways [26], further indicating circadian mechanisms’ importance in melanoma.

By machine learning algorithms, we identified 6 key CRGs (ABCC2, CA14, EGR3, FBXW7, LDHB, and PSEN2) closely associated with melanoma. Among them, 4 key CRGs (ABCC2, CA14, LDHB, PSEN2) were significantly upregulated and 2 key CRGs (EGR3, FBXW7) were significantly downregulated in melanoma tissues. These key CRGs effectively differentiated melanoma from normal tissue and were significantly associated with patient prognosis, suggesting that they may serve as promising diagnostic and prognostic biomarkers. As important components of the circadian rhythm mechanism, these key CRGs are implicated in various biological processes related to tumorigenesis including cell proliferation, apoptosis, cell cycle control, metabolism and signal transduction. ABCC2 (ATP Binding Cassette Subfamily C Member 2), also known as multidrug resistance-associated protein 2 (MRP2), is a protein encoded by the human *ABCC2* gene that mainly participates in intracellular drug transport and metabolism. High levels of ABCC2 expression in melanoma cells and other cancer cells can lead to increased resistance to chemotherapy drugs, thus reducing treatment efficacy and leading to poorer prognosis [27–29]. CA14 (Carbonic Anhydrase 14) is a carbonic anhydrase that is primarily involved in physiological processes including acid-base balance, ion transport, water metabolism and thyroid hormone synthesis. Studies have found that overexpression of CA14 is related to the development and progression of tumors such as breast cancer, prostate cancer, and non-small cell lung cancer [30, 31]. Lee S showed that CA14 can inhibit breast cancer growth and improve breast cancer patient survival by reducing tumor microenvironment acidity and promoting immune infiltration [30]. However, there have been no studies on the role of CA14 in melanoma. This study first discovered CA14 as a risk factor for melanoma development and prognosis. *EGR3* (Early Growth Response Gene 3) is an important circadian rhythm gene mainly expressed in the skin and brain, plays a crucial role in regulating sleep-wake cycles [32–34]. Downregulation of EGR3 has been identified as a risk factor for various types of cancer, including gastric and prostate cancers [35–37]. This study first found EGR3 is downregulated in melanoma, and its low expression significantly impairs survival prognosis in melanoma patients. *FBXW7* (F-box and WD Repeat Domain Containing 7) is a gene encoding a protein that belongs to the F-box protein family, plays an important role in regulating circadian rhythm by enhancing the amplitude of clock gene transcription through regulating the degradation of REV-ERBα [38]. Low expression of FBXW7 is significantly associated with poor prognosis in melanoma patients [39]. Lee et al. [40] found FBXW7 interacts with STAT2 and induces STAT2 instability through the ubiquitination-mediated proteasomal degradation pathway, thereby inhibiting melanoma growth. Aydin et al. [41] showed that FBXW7 mutations and inactivation lead to sustained NOTCH1 activation, promoting angiogenesis in melanoma. FBXW7 can exert anti-melanoma effects by regulating various oncogenes including c-Myc and p53 [42]. FBXW7 also inhibits the MITF/PGC-1α pathway, thereby suppressing melanoma cell proliferation relying on mitochondrial oxidative metabolism [43]. Downregulation of FBXW7 has been observed in breast, gastric and pancreatic cancers, where its low expression associates with unfavorable prognosis [44–46]. As in melanoma, FBXW7 exhibits anti-tumor activities in other cancers by regulating oncogenes such as c-Myc and p53 [47, 48]. Mori et al. [49]. showed that FBXW7 inhibits malignant proliferation and migration of cholangiocarcinoma cells and enhances their sensitivity to cisplatin chemotherapy, by modulating NOTCH1 and MCL1 expression. As a key enzyme in lactate generation during glycolysis, LDHB (lactate dehydrogenase B subunit) encoded by the *LDHB* gene is regulated by the circadian Chrono-Bmal1 pathway, linking circadian disruption with aberrant glucose metabolism and lactate production [50]. LDHB can provide energy for melanoma cell growth through the glycolysis pathway [51], and its high expression could lead to poor prognosis of melanoma patients [52]. LDHB is silenced by promoter methylation in several cancer types, yet overexpressed in most other cancers [53]. LDHB overexpression has been identified as an unfavorable prognostic marker in lung, liver and breast cancers. Knocking down LDHB can inhibit cancer progression in these malignancies [54]. Aside from driving glycolysis, LDHB has also been found to induce pancreatic cancer cell immortalization by activating telomerase [55]. *PSEN2* (Presenilin 2) is a gene encoding the transmembrane proteinase γ-secretase subunit, is involved in regulating the immune system’s function in the circadian rhythm mechanism by regulating the expression level of REV-ERBα [56]. Abnormal high expression of PSEN2 is considered a negative factor in the development and progression of certain cancers such as gastric cancer [57], glioblastoma [58], and ovarian cancer [59]. RNAi-mediated PSEN2 inhibition was found to suppress glioma cell growth and invasion by regulating Nrg1/ErbB signaling [60]. Compared to wild-type mice and control lung cancer cells, PSEN2 knockout mice and PSEN2 knockout lung cancer cells displayed tumor suppressive effects. The mechanisms involve increased DNA binding activity of NF-κB, STAT3 and AP-1, as well as upregulated expression and activity of iPLA2 [61]. Our study is the first to elucidate the significance of PSEN2 in melanoma diagnosis, prognosis and treatment. Overall, these six key CRGs can serve as effective biomarkers for melanoma diagnosis and prognosis evaluation and may become new therapeutic targets for melanoma treatment. Furthermore, to better predict the survival prognosis of melanoma patients, a multivariate Cox prognostic model based on these 6 key CRGs was constructed. The risk score calculated by this model can accurately predict the survival time of melanoma patients. The construction of a nomogram will provide a better predictive tool for the diagnosis and prognosis evaluation of melanoma.

## CONCLUSIONS

In this study, we comprehensively analyzed the roles of circadian rhythm genes in the pathogenesis and prognosis of melanoma using bioinformatics and machine learning methods. We identified 125 CRGs associated with melanoma development, which were mainly involved in biological function like circadian rhythm. Three machine learning algorithms were utilized to identify 6 key CRGs (ABCC2, CA14, EGR3, FBXW7, LDHB, and PSEN2) relevant to diagnosis and prognosis. These key genes were differentially expressed between melanoma and normal skin tissues, and may serve as potential diagnostic and prognostic biomarkers. The risk score model and nomogram constructed based on the key CRGs could effectively predict survival of melanoma patients, providing references for individualized treatment. Our research provides a deeper understanding of the role of circadian rhythm mechanisms in melanoma pathogenesis and the identification of potential therapeutic targets. However, further experimental validation is still needed to determine whether these key genes can become new diagnostic markers or therapeutic targets for melanoma. Overall, our study provides a basis for further clarifying the relationship between melanoma and circadian rhythm and developing novel targeted diagnosis and treatment.

## Supplementary Material

Supplementary Table 1

Supplementary Table 2
